# Is microcystic, elongated, and fragmented pattern of myometrial invasion in endometrioid endometrial carcinoma associated with survival?

**DOI:** 10.55730/1300-0144.5497

**Published:** 2022-07-09

**Authors:** Sultan Deniz ALTINDAĞ, Seyran YİĞİT, Serhat ŞEN

**Affiliations:** 1 Department of Pathology, İzmir Katip Çelebi University Atatürk Research and Training Hospital, İzmir, Turkey; 2 Department of Pathology, Bandırma Training and Research Hospital, Balıkesir, Turkey; 3 Department of Pathology, Faculty of Medicine, İzmir Tınaztepe University, İzmir, Turkey; 4 Department of Gynaecology and Obstetrics, İzmir Katip Çelebi University Atatürk Research and Training Hospital, İzmir, Turkey; 5 Department of Gyneacology and Obstetrics, İstinye University Hospital Medical Park Gaziosmanpaşa, İstanbul, Turkey

**Keywords:** Endometrioid endometrial carcinoma, lymph node metastasis, lymphovascular space invasion, MELF pattern, myometrial invasion, survival

## Abstract

**Background/aim:**

Microcystic, elongated, and fragmented (MELF) pattern is one of the myometrial invasion patterns in endometrioid endometrial carcinoma (EEC), and it has been associated with poor prognostic parameters, especially lymphovascular space invasion (LVSI) and lymph node metastasis (LNM). This study aims to represent the frequency of MELF pattern in EEC and the relationship between MELF pattern with clinicopathological parameters, overall survival (OS), and disease-free survival (DFS).

**Materials and methods:**

In this retrospective cohort study, we examined 233 patients with EEC diagnoses with the result of a total hysterectomy and bilateral salpingo-oophorectomy between 2009 and 2014. The association of MELF pattern with risk factors such as stage, recurrence, and survival was analysed statistically with the comparison of MELF positive and negative cases.

**Results:**

MELF pattern was seen in 21.8% of all cases (51/233) and 23% of grade 1–2 cases (50/217). The MELF pattern showed a significant difference among patients when they were compared according to advanced age (≥60) (p = 0.022), LVSI (p = 0.021), deep myometrial invasion (p < 0.01), LNM (p = 0.027), and advanced FIGO stage (p = 0.043). MELF pattern was a predictive factor of LNM in univariate logistic regression analysis but did not show significance in multivariate analysis. The Kaplan–Meier survival analysis showed that MELF positive cases had reduced OS (66.7% vs 79.7% p = 0.003) and DFS (66.7% vs 77.5% p = 0.017) rates. In the univariate analyses, MELF pattern was an independent prognostic factor on OS and DFS along with other parameters, while it was not observed to maintain this effect in the multivariate analyses.

**Conclusion:**

This study is one of the largest series examining the relationship between MELF pattern of myometrial invasion and survival, and our results represented that the MELF pattern is associated with the worse clinical outcome since it is associated with lymphovascular space invasion, deep myometrial invasion, lymph node metastasis, and reduced overall survival and disease-free survival rates. Although the presence of MELF pattern is not reported in routine practice, it may be used as an indicator that will help predict a worse outcome.

## 1. Introduction

Endometrial cancers are the second most common cancer of gynaecological malignancies after cervical cancer and there are more than 380,000 new cases in 2018 [1]”abstract”: “This article provides a status report on the global burden of cancer worldwide using the GLOBOCAN 2018 estimates of cancer incidence and mortality produced by the International Agency for Research on Cancer, with a focus on geographic variability across 20 world regions. There will be an estimated 18.1 million new cancer cases (17.0 million excluding nonmelanoma skin cancer. Endometrioid endometrial carcinoma (EEC) is the most common subtype and generally has a favourable prognosis [2]. Although most cases are low-grade low-stage, variances in clinical and histopathological features affect both patient prognosis and treatment algorithm [2]. These prognostic features include tumour type and grade, tumour stage, myometrial invasion, cervical involvement, lymph node metastasis (LNM), uterine serosal involvement, adnexal involvement, parametrial invasion, vaginal involvement and lymphovascular space invasion (LVSI) [3]. Recent literature focuses on the patterns of myometrial invasion in EEC as a potential prognostic predictor [4–6]variable five-year survival rates (92%–42%. Microcystic, elongated, and fragmented (MELF) pattern, firstly described by Murray et al., is one of the myometrial invasion patterns in EEC [6,7]they sometimes undergo distinctive changes. These are characterized by outpouchings from typical neoplastic glands that become detached and often lined by flattened epithelium, sometimes appearing as microcysts. The glands may less often become elongated or undergo fragmentation into small solid clusters or single cells. For this constellation of changes, which in aggregate are distinctive, the authors have coined the acronym MELF (microcystic, elongated, fragmented. It is characterized by microcysts lined by cells that often have conspicuous eosinophilic cytoplasm and appeared squamoid, elongated structures, sometimes slit-like lumen, and oedematous or myxoid tissue with retraction artefact and inflammatory cells [7]they sometimes undergo distinctive changes. These are characterized by outpouchings from typical neoplastic glands that become detached and often lined by flattened epithelium, sometimes appearing as microcysts. The glands may less often become elongated or undergo fragmentation into small solid clusters or single cells. For this constellation of changes, which in aggregate are distinctive, the authors have coined the acronym MELF (microcystic, elongated, fragmented. Fragmentation may result in small clusters of cells or single cells, and when only single cells are present, MELF pattern can be easily overlooked. Its recognition is important for the accuracy of measurement of myometrial invasion; if the deep situated MELF pattern is not recognized, it may lead to understaging and thus inadequate treatment [6]. Moreover, the characteristic flattened epithelium of MELF can look like vascular endothelia and can mimic LVSI. MELF pattern has been observed to vary from 9% to 48% in different studies [8,9]. It is reported to be associated with deep myometrial invasion, cervical stromal invasion, LVSI, and LNM [10–18]elongated, and fragmented (MELF. Nevertheless, it remains uncertain whether the presence of the MELF pattern has a clinical significance, because of the limited number of studies. The purpose of this study is to examine the frequency of MELF pattern in EEC and the relationship between MELF pattern with clinicopathological parameters and its effect on survival.

## 2. Materials and methods

Ethics committee approval was obtained from the noninvasive clinical research ethics committee of the hospital for the study (Approval number: 1013, date: 22/10/2020). In this retrospective cohort study, we examined 285 patients with EEC diagnoses with the result of a total hysterectomy and bilateral salpingo-oophorectomy between January, 2009, and December, 2014. Pelvic or pelvic and paraaortic lymphadenectomy was performed in 212 patients. Fifty-two cases confined to the endometrium were excluded from the study. A total of 233 cases with myometrial invasion were reviewed by two pathologists (SDA and SY) on hematoxylin and eosin-stained slides. For each case, 2 to 15 slides of tumour sections were examined. The presence or absence of MELF pattern and histopathological features including tumour grade, depth of myometrial invasion, presence of LVSI, and cervical stromal invasion were evaluated. Patient age, tumour size, presence of LNM, operation date, presence of recurrence and its date, and date of death were obtained from the hospital database. Tumour size data were evaluated in a total of 153 patients since 13 patients had the macroscopically irregular appearance of tumours and 67 patients did not have tumour size data in the archive. Recurrence was confirmed by imaging and/or biopsy. The tumour stage was determined according to the 2009 International Federation of Gynaecology and Obstetrics (FIGO) staging system. Overall survival (OS) was calculated according to the time from the date of operation to the date of last control or death. Disease-free survival (DFS) was defined as the duration between the date of operation and the date of recurrence or last control.

MELF pattern was determined according to the description by Murray et al. [7]they sometimes undergo distinctive changes. These are characterized by outpouchings from typical neoplastic glands that become detached and often lined by flattened epithelium, sometimes appearing as microcysts. The glands may less often become elongated or undergo fragmentation into small solid clusters or single cells. For this constellation of changes, which in aggregate are distinctive, the authors have coined the acronym MELF (microcystic, elongated, fragmented. MELF pattern was characterized by microcysts lined by eosinophilic cells or elongated structures with a squamoid appearance or sometimes slit-like glands, and oedematous or myxoid stroma with retraction artefact and inflammatory cells. On occasion, the fragmentation of glandular structures may result in clusters of cells or individual cells, lying in an oedematous or myxoid stroma. An invasive focus that fulfilled these criteria was considered sufficient to be the identification of the presence of the MELF pattern. MELF pattern was best observed as a myxoid appearance within the myometrium at the low-power examination. When the myxoid stroma is observed at the edge of the tumour, eosinophilic tumour cells and microcystic or fragmented glands intermingled with inflammation approve the presence of the MELF pattern at the high-power examination.

### 2.1. Statistical analysis

The association of MELF pattern with age, tumour size, tumour grade, depth of myometrial invasion, presence of LVSI, cervical stromal invasion, LNM, recurrence, and FIGO stage was analysed statistically to compare MELF positive and negative cases in both all cases and grade 1–2 cases. For statistical analysis, IBM SPSS for Windows v. 24.0 (IBM Corp., Armonk, NY) was used. The normal distribution of the data was evaluated by analytical methods (The Kolmogorov–Smirnov and Shapiro–Wilk tests). To compare qualitative and quantitative parameters, t-test, the Mann–Whitney U test, and Pearson’s chi-squared test were used. Univariate and multivariate logistic regression models were performed to see the predictive factors of LNM. OS and DFS were calculated with the Kaplan–Meier method and the effects of various variables on survival were evaluated by using the log-rank test. Multivariate analysis based on the results of univariate analysis of factors affecting survival was performed by Cox regression analysis and the proportional hazard assumption (hazard ratio, HR) was calculated. The study was conducted at a 95% confidence interval (CI). For statistical significance, p was determined <0.05.

## 3. Results

### 3.1. MELF pattern in endometrioid endometrial carcinoma

**Figure 1:**
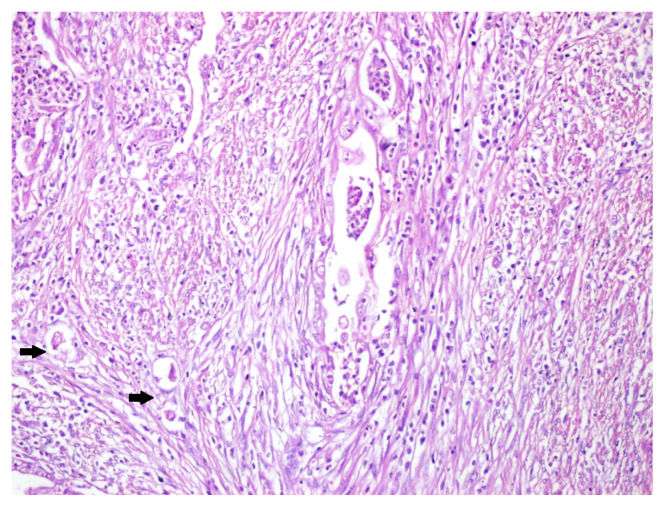
Fragmented, irregularly shaped glands located within fibromyxoid stroma, lumen filled with histiocytic and neutrophilic infiltration; note that individual neoplastic cells with retraction artefact in the lower-left corner (arrows); HE x400.

**Figure 2:**
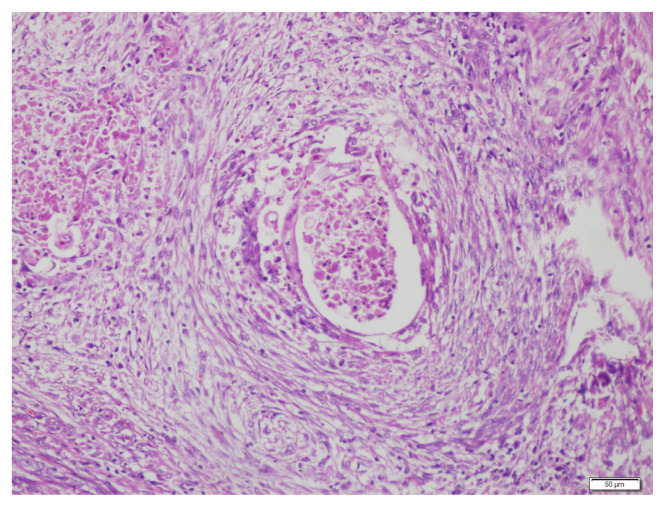
Microcystic, elongated, and fragmented tumour gland next to conventional tumour glands; fragmented, eosinophilic gland lined by attenuated cells, its lumen containing infiltration, and surrounded by fibromyxoid stroma; HE x400.

There were 233 EEC cases included. MELF pattern was detected in 51 cases (21.8%) (Figure 1). The clinicopathological comparison of MELF positive and negative cases in all cases is presented in Table 1. MELF pattern was frequently observed in the deepest extent of invasion. While in 6 cases MELF pattern was considered a pure invasion pattern, it was seen as a predominant type in 3 cases. In the remaining 42 cases, MELF pattern was observed as a secondary pattern (Figure 2).

MELF presence showed significantly higher frequency in patients older than 60 years (p = 0.022), LVSI (p = 0.021), deep myometrial invasion (p < 0.01), LNM (p = 0.027), and advanced FIGO stage (p = 0.043). The results of univariate and multivariate logistic regression models are given in Table 2. Based on the results of univariate logistic regression analysis, age and grade were not included in multivariate analysis.

During the follow-up, 3 MELF-positive patients (5.9%) and 18 MELF-negative patients (9.9%) had a recurrence. The recurrence site of MELF-positive cases was vaginal in 2 patients and vaginal and distant in 1 patient. The recurrence site of MELF-negative cases was vaginal in 1 patient, pelvic in 2 patients, abdominal in 2 patients, distant in 9 patients, vaginal and distant in 2 patients, vaginal and pelvic in 1 patient, and vaginal, pelvic, and distant in 1 patient.

### 3.2. MELF pattern in grade 1 and 2 endometrioid endometrial carcinoma

Clinicopathologic characteristics of all grade 1 and 2 EEC cases (n = 217) with the comparison of MELF-positive and -negative cases are shown in Table 3. MELF pattern was detected in 50 cases (23%). MELF presence showed significantly higher frequency in patients older than 60 years (p = 0.019), LVSI (p = 0.008), deep myometrial invasion (p < 0.01), LNM (p = 0.019), and advanced FIGO stage (p = 0.032). The results of univariate and multivariate logistic regression models are given in Table 4. Based on the results of univariate logistic regression analysis, age was not included in multivariate analysis.

### 3.3. MELF pattern and survival

**Figure 3:**
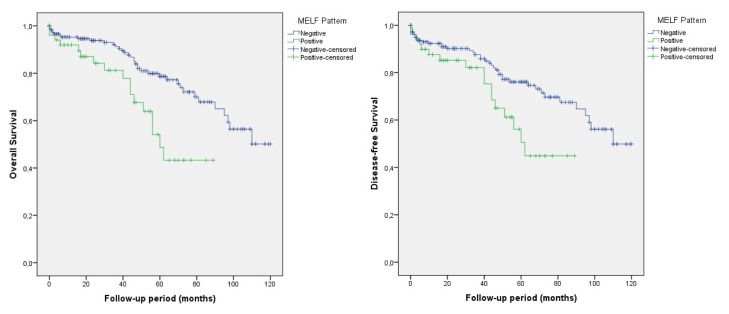
The Kaplan–Meier analysis showing overall survival and disease-free survival for MELF-positive and -negative patients in all cases (p = 0.003, p = 0.017).

**Figure 4:**
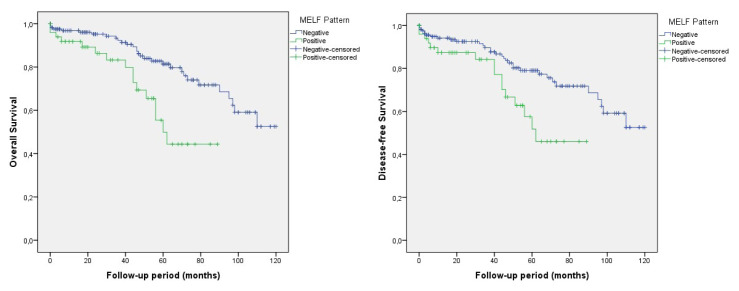
Kaplan-Meier analysis showing overall survival and disease-free survival for MELF-positive and -negative patients in grade1 and 2 cases (p = 0.001, p = 0.008).

Of all 233 patients, the median follow-up period was 44 months. In the Kaplan–Meier survival analysis, MELF-positive cases were found to have reduced OS and DFS rates compared to negative cases, and it was statistically significantly associated with OS (66.7% vs 79.7%, p = 0.003), and DFS (66.7% vs 77.5%, p = 0.017) by log-rank test (Figure 3). Analysis of grade 1 and 2 cases showed that MELF-positive cases with reduced OS and DFS rates when compared to negative cases (68.0% vs 82.0%, p = 0.001; 68.0% vs 80.2%, p = 0.008) (Figure 4).

The results of univariate and multivariate analyses of prognostic parameters affecting OS and DFS are given in Tables 5 and 6. Based on the results of the univariate analyses in Cox regression model with DFS, age was not included in multivariate analyses.

## 3. Discussion

This large series of 233 endometrioid endometrial carcinomas examining the relationship between MELF pattern of myometrial invasion and clinicopathological parameters shows that MELF pattern is associated with LVSI, deep myometrial invasion, and LNM in both all cases and grade 1–2 cases. Moreover, the presence of MELF pattern is associated with reduced overall survival and disease-free survival rates. MELF pattern was described by Murray et al. in 2003, and since then several studies have examined the prognostic value of MELF pattern [10–16,18–20]. In this study, the MELF pattern was observed in 21.8% of all cases and 23% of grade 1–2 cases. Our prevalence is within the reported frequency of MELF pattern in different studies which ranges from 9% to 48% [9–18].

Although there are studies in the literature that have not found a relationship between the presence of MELF pattern and age [11,12,14], both Naki et al. and Eriksson et al. reported a significant relationship between higher mean age and MELF pattern [10,21]. In our study, there was no significant relationship when comparing the mean ages of MELF-positive and -negative cases. However, a statistically significant correlation was found when the age was divided into two groups as under and over 60.

Many studies in the literature have examined MELF pattern as a predictive factor of LNM and the relation between these two entities [9,11–19,22]. While Pelletier et al., Joehlin-Price et al., and Han et al. reported an association between LNM and MELF pattern in only grade 1 EEC cases, Sanci et al. and Euscher et al. reported it in grade 1–2 EEC cases [9,13,15,17,22]. We examined both all EEC cases and grade 1–2 cases in this study and showed a statistically significant relationship between LNM and MELF pattern similar to the results in the literature [11,12,14,16,18]. When we evaluated positive lymph nodes as isolated pelvic, isolated paraaortic, and dual involvement, it was observed that there was no significant difference between MELF-positive and -negative cases. In their study where all cases harboured LVSI, that MELF pattern is independently associated with an increased rate of LNM [19]. Both Han et al. and Euscher et al. reported in their studies that MELF pattern was a predictive factor of LNM in univariate analysis, but not persisted in multivariate analysis [9,22]. Moreover, Eriksson et al. conducted a study to describe the sonographic features of MELF pattern, and a significant relationship was found between the presence of MELF pattern and LNM, which proved significant in univariate analysis but not in multivariate analysis [21]. While Dogan Altunpulluk et al. found that MELF pattern was a significant predictor of LNM on multivariate analysis, Sanci et al. found this result only among grade 1–2 EEC cases [14,15]. Pavlakis et al. divided their cases into 2 groups according to whether lymph node sampling was performed or not and stated that MELF pattern significantly increased the possibility of lymph node metastasis by logistic regression analysis in the group which underwent lymphadenectomy [16]. Espinosa et al. reported that MELF pattern was not associated with LNM by logistic regression analysis [11]. In our study, the presence of MELF pattern, LVSI, deep myometrial invasion, and cervical stromal invasion showed a significant correlation with LNM in univariate analyses; only LVSI, deep myometrial invasion, and cervical stromal invasion showed significance in multivariate analyses.

Although lymphovascular invasion is an uncommon finding in EEC, it is the strongest independent prognostic factor for LNM and overall survival [23,24]. Many studies have analysed the relationship between LVSI and the presence of MELF pattern and reported that LVSI is likely to be associated with MELF pattern [10–12,14,18,20]. In the current study, we also reported a significant relationship following the literature. In previous studies, it was mentioned that LVSI and MELF pattern might be morphologically confused and using D2–40 and cytokeratin staining will help with this distinction [14,20]. When the characteristic elongated, slit-like glandular epithelium of MELF changes resembles vascular endothelium in some cases, the presence of the fibromyxoid stromal changes favours MELF pattern.

Myometrial invasion is an indispensable component of FIGO staging system and an indicator of poor prognosis [3]. Most studies noted a statistically significant result between the depth of myometrial invasion and the presence of the MELF pattern [10,12,14,21], whereas two studies found no difference [11,15]. In the present study, we revealed a significant relationship between MELF pattern and deep myometrial invasion. MELF pattern is frequently observed in the deepest extent of invasion [7,20]. The inconspicuous appearance of the single cells or small clusters of cells can lead to undetectability, and its recognition is important for the accuracy of the depth of myometrial invasion. Furthermore, when we examine the relationship between FIGO staging and the presence of the MELF pattern, a significant relationship was found between the presence of MELF and the advanced stage in this study similar to previous studies [12,14,21].

Zinovkin et al. reported that the observed survival rate of patients with EEC was significantly lower in MELF positive patients when compared with MELF negative patients, and in multivariate analysis, MELF pattern was an independent prognostic factor for survival [25]. Sanci et al., in their study of grade 1–2 EEC cases, revealed that the effect of the MELF pattern on OS was significant whereas there were no significant differences in DFS; also, MELF pattern has not associated with OS in multivariate analysis [15]. In the systematic review of Prodromidou et al., no difference was reported in DFS and disease-specific survival in the studies observing the relationship between MELF pattern and survival; and as a conclusion, they had commented that the implication of MELF pattern in survival was ill-determined [8]. In the present study, according to the Kaplan–Meier analysis, MELF-positive cases were significantly associated with reduced OS and DFS in both all EEC cases and grade 1–2 cases. In the univariate analyses, the presence of MELF pattern was observed to be an independent prognostic factor on OS and DFS along with other parameters (age, grade, LVSI, myometrial invasion, cervical stromal invasion, stage), while it was not observed to maintain this effect on the multivariate analyses.

There are studies in the literature that conclude the relationship between the presence of MELF and grade differently. While 2 studies reported that MELF was associated with high-grade tumours [10,26], the more common observation in the previous studies was that MELF pattern was associated with low-grade tumours [12,14,16,20]. In this study, most of the cases were low-grade (50/51) and grade 3 was only seen in 1 case, and no significant relationship was found between the presence of MELF and grade.

Multicentre studies which examined the relationship between the presence of MELF pattern and recurrence have reported an association between them [27,28]. Our results indicated that only 3 MELF-positive cases had recurrence, which was vaginal in 2 patients and vaginal and distant in 1 patient. No significant relation was found between MELF pattern and recurrence in our study, but in this cohort, very few MELF-positive cases had recurrence. In conclusion, we analysed the association between MELF pattern and clinicopathological parameters and its effect on survival in 233 EEC cases with myometrial invasion in all EEC cases and grade 1–2 cases. Our results represent that MELF pattern was associated with advanced age (≥60), lymphovascular space invasion, deep myometrial invasion, lymph node metastasis, and advanced FIGO stage. In the survival analysis, MELF-positive cases were found to have reduced overall and disease-free survival rates compared to negative cases, yet it has no effect on OS and DFS in the multivariate analyses. Our study has limitations due to its retrospective nature and limitation to a single tertiary care centre. The small number of recurrences in this cohort decreases the strength of the analysis. Albeit its limitations, we believe that this study is remarkable as it is one of the largest series examining the relationship between MELF pattern of myometrial invasion and survival in both all EEC cases and grade 1–2 cases. Although the prognostic significance of MELF pattern has not been consistently demonstrated, it may enter into our routine pathology practice in the near future as a useful indicator of a worse outcome.

## Figures and Tables

**Table 1:** The clinicopathological comparison of MELF-positive and -negative cases in all cases.

Present		MELF pattern (n (%))		Total (n (%))	p-value
		Absent			
**Total cases**		51 (21.8%)	182 (78.1%)	233 (100%)	
**Age (years) (mean ± SD)**		62.5 ± 9.1	60.3 ± 9.1	60.8 ± 9.1	0.095 a
**Tumour size (cm) (mean ± SD)**		3.2 ± 1.6 (n = 33)	3.2 ± 1.9 (n = 120)	3.2 ± 1.8 (n = 153)	0.571 b
**Age**	<60	16 (31.4%)	90 (49.5%)	106 (45.5%)	**0.022** c
	≥60	35 (68.6%)	92 (50.5%)	127 (54.5%)	
**Grade**	1	28 (54.9%)	113 (62.1%)	141 (60.5%)	0.089 c
	2	22 (43.1%)	54 (29.7%)	76 (32.6%)	
	3	1 (2.0%)	15 (8.2%)	16 (6.9%)	
**Lymphovascular space invasion**	Absent	39 (76.5%)	162 (89%)	201 (86.3%)	**0.021** c
	Present	12 (23.5%)	20 (11%)	32 (13.7%)	
**Myometrial invasion**	<50%	18 (35.3%)	121 (66.5%)	139 (59.7%)	**0.000** c
	≥50%	33 (64.7%)	61 (33.5%)	94 (40.3%)	
**Cervical stromal invasion**	Absent	42 (82.4%)	164 (90.1%)	206 (88.4%)	0.126 c
	Present	9 (17.6%)	18 (9.9%)	27 (11.6%)	
**Lymphadenectomy**	Pelvic	17 (37.0%)	58 (34.9%)	75 (35.4%)	0.800 c
	Pelvic and paraaortic	29 (63%)	108 (65.1%)	137 (64.6%)	
**Lymph node metastasis**	Absent	36 (78.3%)	150 (90.4%)	186 (87.7%)	**0.027** c
	Present	10 (21.7%)	16 (9.6%)	26 (12.3%)	
**Number of positive lymph nodes**	Isolated pelvic	4 (40%)	10 (62.5%)	14 (53.8%)	0.150 c
	Isolated paraaortic	2 (20%)	0 (0%)	2 (7.7%)	
	Pelvic and paraaortic	4 (40%)	6 (37.5%)	10 (38.5%)	
**Recurrence**	Absent	48 (94.1%)	164 (90.1%)	212 (91%)	0.377 c
	Present	3 (5.9%)	18 (9.9%)	21 (9%)	
**FIGO stage**	IA	17 (33.3%)	117 (64.3%)	134 (57.5%)	
	IB	18 (35.3%)	38 (20.9%)	56 (24%)	
	II	6 (11.8%)	10 (5.5%)	16 (6.9%)	
	IIIA	1 (2%)	1 (0.5%)	2 (0.9%)	
	IIIB	0 (0%)	1 (0.5%)	1 (0.4%)	
	IIIC1	3 (5.9%)	8 (4.4%)	11 (4.7%)	
	IIIC2	6 (11.8%)	3 (1.6%)	9 (3.9%)	
	IVA	0 (0%)	1 (0.5%)	1 (0.4%)	
	IVB	0 (0%)	3 (1.6%)	3 (1.3%)	
**FIGO stage**	Stages I-II	41 (80.4%)	165 (90.7%)	206 (88.4%)	**0.043** c
	Stages III-IV	10 (19.6%)	17 (9.3%)	27 (11.6%)	

MELF: Microcystic, elongated, and fragmented, n: number, SD: standard deviation, FIGO: Federation of Gynaecology and Obstetrics,

a t-test,

b the Mann–Whitney U test,

c Pearson’s chi-squared test.

**Table 2:** Results of univariate and multivariate analyses in the logistic regression model in all cases.

	Univariate analysis			Multivariate analysis		
	HR	95% CI	p	HR	95% CI	p
**Age (≥60)**	2.064	0.855–4.983	0.107	-	-	-
**Grade (G1–G2/ G3)**	1.109	0.236–5.218	0.896	-	-	**-**
**MELF pattern**	2.604	1.091–6.215	**0.031**	1.087	0.324–3.651	0.892
**Lymphovascular space invasion**	35.795	12.755–100.454	**0.000**	24.231	7.685–76.401	**0.000**
**Myometrial invasion**	14.615	4.227–50.531	**0.000**	6.476	1.569–26.727	**0.010**
**Cervical stromal invasion**	7.125	2.755–18.426	**0.000**	4.074	1.144–14.515	**0.030**

HR: Hazard ratio, CI: Confidence interval

**Table 3:** The clinicopathological comparison of MELF-positive and -negative cases in grade 1 and 2 cases.

Present		MELF Pattern (n (%))		Total (n (%))	*p* value
		Absent			
**Total cases**		50 (23%)	167 (77%)	217 (100%)	
**Age (years) (mean ± SD)**		62.2 ± 8.8	60 ± 9.2	60.5 ± 9.1	0.515 a
**Tumour size (cm) (mean ± SD)**		3.3 ± 1.6 (n = 32)	3.0 ± 1.7 (n = 107)	3.1 ± 1.7 (n = 139)	0.256 b
**Age**	<60	16 (32%)	85 (50.9%)	101 (46.5%)	**0.019** c
	≥60	34 (68%)	82 (49.1%)	116 (53.5%)	
**Lymphovascular space invasion**	Absent	38 (76%)	151 (90.4%)	189 (87.1%)	**0.008** c
	Present	12 (24%)	16 (9.6%)	28 (12.9%)	
**Myometrial invasion**	<50%	18 (36%)	117 (70.1%)	135 (62.2%)	**0.000** c
	≥50%	32 (64%)	50 (29.9%)	82 (37.8%)	
**Cervical stromal invasion**	Absent	42 (84%)	153 (91.6%)	195 (89.9%)	0.117 c
	Present	8 (16%)	14 (8.4%)	22 (10.1%)	
**Lymphadenectomy**	Pelvic	16 (35.6%)	56 (36.8%)	72 (36.5%)	0.875 c
	Pelvic and paraaortic	29 (64.4%)	96 (63.2%)	125 (63.5%)	
**Lymph node metastasis**	Absent	3 (77.8%)	138 (90.8%)	173 (87.8%)	**0.019** c
	Present	10 (22.2%)	14 (9.2%)	24 (12.2%)	
**Number of positive lymph nodes**	Isolated pelvic	4 (40%)	9 (64.3%)	13 (54.2%)	0.177 c
	Isolated paraaortic	2 (20%)	0 (0%)	2 (8.3%)	
	Pelvic and paraaortic	4 (40%)	5 (35.7%)	9 (37.5%)	
**Recurrence**	Absent	47 (94%)	153 (91.6%)	200 (92.2%)	0.582 c
	Present	3 (6%)	14 (8.4%)	17 (7.8%)	
**FIGO stage**	IA	17 (34%)	113 (67.7%)	130 (59.9%)	
	IB	18 (36%)	31 (18.6%)	49 (22.6%)	
	II	5 (10%)	8 (4.8%)	13 (6%)	
	IIIA	1 (2%)	1 (0.6%)	2 (0.9%)	
	IIIB	0 (0%)	1 (0.6%)	1 (0.5%)	
	IIIC1	3 (6%)	7 (4.2%)	10 (4.6%)	
	IIIC2	6 (12%)	2 (1.2%)	8 (3.7%)	
	IVA	0 (0%)	1 (0.6%)	1 (0.5%)	
	IVB	0 (0%)	3 (1.8%)	3 (1.4%)	
**FIGO stage**	Stages I-II	40 (80%)	152 (91%)	192 (88,5%)	**0.032** c
	Stages III-IV	10 (20%)	15 (9%)	25 (11.5%)	

MELF: Microcystic, elongated, and fragmented, n: number, SD: standard deviation, FIGO: Federation of Gynaecology and Obstetrics,

a t-test,

b the Mann–Whitney U test,

c Pearson’s chi-squared test.

**Table 4:** Results of univariate and multivariate analysis in the logistic regression model in all cases.

	Univariate analysis			Multivariate analysis		
	HR	95% CI	p	HR	95% CI	p
**Age (≥60)**	1.888	0.768–4.641	0.166	-	-	**-**
**MELF pattern**	2.816	1.154–6.874	**0.023**	0.981	0.281–3.426	0.977
**Lymphovascular space invasion**	32.600	11.271–94.290	**0.000**	22.563	6.828–74.562	**0.000**
**Myometrial invasion**	15.018	4.297–52.488	**0.000**	7.563	1.817–31.470	**0.005**
**Cervical stromal invasion**	6.708	2.391–18.821	**0.000**	4.034	1.047–15.536	**0.043**

HR: Hazard ratio, CI: Confidence interval

**Table 5:** Results of univariate and multivariate analyses in Cox regression model with overall survival and disease-free survival in all cases.

	Univariate analysis			Multivariate analysis		
	HR	95% CI	p	HR	95% CI	p
***Overall survival***						
**Age (≥60**)	1.982	1.131–3.472	**0.017**	1.480	0.804–2.725	0.208
**Grade (G1–G2/ G3)**	2.477	1.167–5.260	**0.018**	2.624	1.153–5.972	**0.021**
**FIGO (I-II/ III-IV)**	5.711	3.235–10.084	**0.000**	3.459	1.438–8.320	**0.006**
**MELF pattern**	2.423	1.337–4.390	**0.004**	1.268	0.633–2.541	0.503
**Lymphovascular space invasion**	3.703	2.072–6.619	**0.000**	0.982	0.408–2.362	0.967
**Myometrial invasion**	4.510	2.512–8.097	**0.000**	2.252	1.162–4.365	**0.016**
**Cervical stromal invasion**	5.257	2.846–9.709	**0.000**	2.687	1.375–5.254	**0.004**
***Disease-free survival***						
**Age (≥60)**	1.701	0.998–2.897	0.051	-	-	-
**Grade (G1–G2/ G3)**	2.860	1.402–5.834	**0.004**	3.006	1.372–6.584	**0.006**
**FIGO (I–II/ III–IV)**	6.253	3.628–10.779	**0.000**	3.840	1.713–8.606	**0.001**
**MELF pattern**	2.001	1.120–3.577	**0.019**	1.085	0.555–2.122	0.811
**Lymphovascular space invasion**	3.688	2.104–6.467	**0.000**	1.006	0.450–2.248	0.989
**Myometrial invasion**	4.205	2.407–7.345	**0.000**	2.268	1.196–4.300	**0.012**
**Cervical stromal invasion**	5.205	2.865–49.454	**0.000**	2.482	1.286–4.790	**0.007**

HR: Hazard ratio, CI: Confidence interval

**Table 6:** Results of univariate and multivariate analysis in Cox regression model with overall survival and disease-free survival in grade 1 and 2 cases.

	Univariate analysis			Multivariate analysis		
	HR	95% CI	p	HR	95% CI	p
***Overall survival***						
**Age (≥60**)	1.895	1.039–3.459	**0.037**	0.658	0.336–1.287	0.221
**FIGO (I–II/ III–IV)**	5.987	3.252–11.023	**0.000**	0.256	0.103–0.637	**0.003**
**MELF pattern**	2.721	1.444–5.127	**0.002**	0.753	0.365–1.553	0.443
**Lymphovascular space invasion**	3.542	1.877–6.684	**0.000**	0.795	0.310–2.043	0.634
**Myometrial invasion**	4.286	2.303–7.977	**0.000**	2.280	1.125–4.619	**0.022**
**Cervical stromal invasion**	4.441	2.237–8.820	**0.000**	0.438	0.210–0.910	**0.027**
***Disease-free survival***						
**Age (≥60)**	1.719	0.965–3.062	0.066	-	-	-
**FIGO (I-II/ III-IV)**	6.767	3.768–12.151	**0.000**	3.937	1.711–9.060	**0.001**
**MELF pattern**	2.256	1.217–4.184	**0.010**	1.093	0.544–2.195	0.803
**Lymphovascular space invasion**	3.691	1.999–6.817	**0.000**	0.883	0.368–2.115	0.779
**Myometrial invasion**	4.266	2.339–7.780	**0.000**	2.570	1.297–5.091	**0.007**
**Cervical stromal invasion**	4.637	2.381–9.029	**0.000**	2.145	1.050–4.380	**0.036**

HR: Hazard ratio, CI: Confidence interval
